# Prolonged exposure to low oxygen improves hypoxia tolerance in a freshwater fish

**DOI:** 10.1093/conphys/coz058

**Published:** 2019-11-28

**Authors:** Kayla L Gilmore, Zoe A Doubleday, Bronwyn M Gillanders

**Affiliations:** Southern Seas Ecology Laboratories, School of Biological Sciences and Environment Institute, University of Adelaide, SA 5005, Australia

**Keywords:** Aerobic ability, Murray cod, *P*_crit_, physiological threshold, respirometry

## Abstract

Persistent hypoxic or low-oxygen conditions in aquatic systems are becoming more frequent worldwide, causing large-scale mortalities to aquatic fauna. It is poorly understood, however, whether species can acclimate to long-term hypoxic conditions. In two experiments, we exposed juvenile freshwater fish (Murray cod, *Maccullochella peelii*) to low-oxygen conditions and investigated acclimation effects. Experiment 1 determined how responses could be modified by exposure to different temperatures (20, 24 and 28°C) and oxygen conditions (control 6–8 mgO_2_ L^−1^ and low-oxygen 3–4 mgO_2_ L^−1^) over 30 days. Experiment 2 determined the acclimation ability of fish exposed to two temperatures (20 and 28°C) and low-oxygen conditions (3–4 mgO_2_ L^−1^) for three different acclimation periods (7, 14 and 30 days). Responses were measured by determining critical oxygen tension (*P*_crit_), loss of equilibrium and aerobic capacity using resting respirometry. In experiment 1, resting oxygen requirements were negatively affected by long-term low-oxygen exposure except at the highest temperature (28°C). However, long-term acclimation in low-oxygen improved tolerance as measured by loss of equilibrium but not *P*_crit_. In experiment 2, fish could tolerate lower oxygen levels before reaching loss of equilibrium after 7 days acclimation, but this declined overtime. Murray cod were most tolerant to low-oxygen at the lowest temperature (20°C) and shortest exposure time (7 days). Extended low-oxygen exposure resulted in reduced aerobic capacity of fish particularly at the lowest temperature. While prior exposure to low-oxygen may allow fish to cope with hypoxic conditions better in the long-term, acclimation time was inversely related to tolerance, suggesting that resistance to hypoxia might decrease as a function of exposure time. Our study fills a much-needed gap in our understanding of how freshwater species acclimate to hypoxia, and in particular, how exposure to prolonged periods of low-oxygen and elevated temperatures affect organisms physiologically.

## Teaser

Lay summary

It is poorly understood whether fish can acclimate to prolonged low-oxygen conditions (or hypoxia). Our study shows that prior long-term exposure to low-oxygen conditions improves tolerance to low-oxygen in a freshwater fish. The results of our study aid our understanding of long-term responses of freshwater fish to low-oxygen to hypoxic events.

## Introduction

Hypoxia can be fatal to many organisms including mammals, birds, fish, reptiles and invertebrates (Hermes-Lima and Zenteno-Savín 2002; Bickler and Buck 2007; Ramirez *et al.* 2007). However, countless species have become adapted to periods of hypoxia ranging from hours to months (Hermes-Lima and Zenteno-Savín 2002; Bickler and Buck 2007). Extensive research has shown many of the biochemical and physiological mechanisms that allow animals to endure oxidative stress (for reviews see: Hochachka and Lutz 2001; Hermes-Lima and Zenteno-Savín 2002; Bickler and Buck 2007; Ramirez *et al.* 2007). However, the focus has mostly been on the cellular response pathways, protein synthesis, gene expression and metabolic constraints of organisms under short-term (hours) exposure to hypoxia with few studies considering long-term exposure to hypoxia and the potential for a species to acclimate to low-oxygen conditions.

Fishes have adapted to almost all aquatic habitats on Earth and can be found living in some of the most extreme environments, yet they are also considered to be some of the most sensitive taxa to hypoxia (Vaquer-Sunyer and Duarte 2008; Gräns *et al.* 2014). However, the ability of fish to acclimate and adapt to hypoxia has received little attention. Furthermore, fish species have not evolved to tolerate all conditions simultaneously and often exhibit species-specific responses, for example, certain species tolerate temperatures from −2°C in the polar regions to +44°C in African lakes and from 1 mgO_2_ L^−1^ of oxygen to 8 mgO_2_ L^−1^ (Gräns *et al.* 2014). Phylogenetic comparisons of fish species show that hypoxia tolerance has arisen independently many times amongst different lineages and geographical locations (Hochachka and Lutz 2001; Mandic *et al.* 2009; Killen *et al.* 2016). Research on the effects of hypoxia is mainly focussed on marine and estuarine species, with considerably less attention given to freshwater species (Diaz and Rosenberg 2011; Rogers *et al.* 2016). The paucity of research examining acclimation of fish, particularly for freshwater species, highlights the need for additional research.

Resting respirometry is one method used to determine the thermal tolerance of fish, and more recently, hypoxia tolerance (Roche *et al.* 2013; Nelson and Lipkey 2015). Thermal tolerance, in addition to hypoxia tolerance, is an important consideration for physiological and behavioural studies as it affects both oxygen demand and the amount of dissolved oxygen available in the water (McBryan *et al.* 2013; Claireaux and Chabot 2016). For example, every 10°C increase in temperature results in a 10 to 20% decrease in dissolved oxygen (Farrell and Richards 2009). Therefore, fish that experience a broad thermal range, such as freshwater and estuarine species, will be strongly influenced by changes in oxygen levels and temperature. Resting respirometry represents an ideal experimental solution to predict and test organism responses to multiple levels of environmental conditions like hypoxia and temperature.

Rapid changes in water partial oxygen pressure (PO_2_) can have dire consequences on aquatic fauna as their capacity to respond to hypoxia is dictated by functioning physiological and biochemical systems in place at the time of exposure (Farrell and Richards 2009). If fish are unable to extract oxygen efficiently from the environment during progressive hypoxia exposure, they become less tolerant (Farrell and Richards 2009). Some fish may be able to acclimate by initiating physiological and biochemical changes to enhance body function and extend survival; however, the temporal scope of this resistance is poorly understood. Furthermore, as increased temperatures result in a reduction in available dissolved oxygen in the water, temperature may also diminish the resistance of fish to hypoxia, as it increases metabolism in ectotherms. The temporal resistance of fish to hypoxia and their acclimation ability have been largely overlooked in the literature.

We investigated whether prior exposure to hypoxia or low-oxygen could improve the tolerance of freshwater fish to hypoxic conditions. We used Murray cod (*Maccullochella peelii*), a susceptible freshwater fish native to the Murray Darling Basin, an extensive river system that is frequently influenced by natural and anthropogenic hypoxic events (for further species information see SI). First, we tested how Murray cod responded to long-term low-oxygen exposure at different temperatures, and then we tested how low oxygen exposure for different lengths of time and different temperatures modified fish responses. We measured aerobic capacity, the critical oxygen limit of fish (*P*_crit_) and loss of equilibrium to determine if there was an acclimation response to low-oxygen. We predicted that (a) the combination of high temperatures and prolonged exposure to low-oxygen would exacerbate the effects of hypoxia and limit a fish’s ability to acclimate and (b) we predicted that duration of exposure to low-oxygen would alter the acclimation capacity of fish such that, longer duration of exposure would decrease acclimation capacity.

## Methods

### Experimental design

Juvenile Murray cod (*M. peelii*), approximately 55 mm in length and 1.5 g average body mass, were obtained from aquaculture stock from the NSW Hatchery Quality Assurance Scheme accredited Silverwater Native Fish Hatchery, Grong Grong, NSW, in March 2015. Fish were kept in 250 L holding tanks at 20°C at the University of Adelaide before being assigned to 20 L tanks for the experimental treatments. All tanks (250 L holding tanks and 20 L experimental tanks) were filled using aged (dechlorinated) tap water and aerated and filtered for the duration of the experiments; evaporation was minimized using plexiglass lids. Fish were fed hatchery pellet food until satiation with excess food siphoned out an hour after feeding. Room temperature was maintained at 20°C and fish were exposed to a 12:12 hr light:dark cycle. Water changes of 25% were made daily and water quality was monitored every second day for temperature, oxygen levels and saturation, pH, ammonia and nitrite.

For Experiment 1, we exposed fish for 30 days to two oxygen treatments (normoxic 6-8 mgO_2_ L^−1^ or 12–14 kPa and low-oxygen 3–4 mgO_2_ L^−1^ or 7–9 kPa) and three temperature treatments reflective of the species’ natural thermal range; (20, 24 and 28°C; Lintermans 2007) in an orthogonal design (n = 6 treatments). Each treatment was duplicated resulting in 12 tanks. Murray cod experience temperatures ranging from 4 to 34°C across their natural range; temperatures chosen reflect those most likely to be experienced at the higher end of the thermal range as this range would be most affected by low-oxygen exposure (Lintermans 2007). Experiment 2 consisted of three acclimation treatments (7, 14 and 30 days exposure to low-oxygen conditions 3–4 mgO_2_ L^−1^ or 7–9 kPa) and two temperature treatments (20 and 28°C) again with duplicate tanks in an orthogonal design (*n* = 6 treatments, 3 acclimation durations × 2 temperatures). However, only 8 new tanks (including duplicates) were used for Experiment 2 as fish from low-oxygen treatments in Experiment 1 at 20 and 28°C were used to measure 30 days of acclimation. Experiment 2 was run following the completion of Experiment 1. Fish were randomly assigned to tanks (20 L), with about 11 fish per tank. Fish in treatments ≥24°C were acclimated 2°C per day by adjusting the submersible aquarium heaters. To ensure survival of fish during experimentation, low-oxygen levels (3–4 mgO_2_ L^−1^ or 7–9 kPa) were higher than the globally accepted tolerance limit of 2 mgO_2_ L^−1^, which is believed to frequently result in mortality of aquatic species (Vaquer-Sunyer and Duarte 2008).

The experiment was designed to provide long-term exposure yet still subject fish to low-oxygen conditions and minimize mortality of individuals (i.e. sub-lethal treatments) while allowing physiological responses to be tested. For the low-oxygen treatments, we developed a simple method to deoxygenate the water (Gilmore *et al.* 2018). Nitrogen gas (9 L/min split across 3 food grade G-Class nitrogen cylinders, at 3 L/min/cylinder) was mixed with 9 L/min of air (from an air compressor) in a loosely sealed 35 L mixing chamber. Two electric air pumps running within the mixing chamber pumped the mixed gas into relevant individual tanks using air hosing (of equal distance) connected to single air stones (of the same size) with a combined flow rate of ~ 18 L/min. Plexiglass lids covered all tanks to minimize turbulence and limit diffusion of surrounding atmospheric air. Oxygen levels in all 14 ‘low-oxygen’ tanks could be simultaneously controlled for extended periods (Experiment 1 low-oxygen tanks = 6, control = 6, Experiment 2 low-oxygen tanks = 8, the additional 4 tanks required in this analysis were from Experiment 1).

Length and weight of each fish were measured at the completion of the experiments and used to calculate a simple condition index, Fulton’s *K*, which assumes that the weight of a fish is proportional to the cube of its length:

$$K=100\left(W/L^{3}\right).$$

Where *W* is body wet weight (g) and L the total standard length (McMaster and Bond 2008); 100 is used to bring the factor close to a value of one. Fulton’s *K* condition index is widely used in fish biology studies to describe the condition of the individual and has been used in our experiment to show how the condition may have changed in the different treatments (Nash *et al.* 2006).

Experimental treatment conditions were maintained consistently for all experimental periods (Tables S1 and S2). Fish lengths and weights showed little variation amongst treatments (Table S1).

### Intermittent respirometry

Following 7, 14 or 30 days exposure, fish were fasted for 24 hrs prior to experimental trials to evacuate the digestive tract so that only oxygen consumption rates (Ṁo_2_) were recorded. Fasting fish were held in an isolated container in the larger water bath where respirometry experiments were conducted, to ensure there was no shock experienced prior to being placed in resting chambers. Twelve fish per treatment were randomly selected and subjected to respirometry experiments.

Three fish were tested simultaneously using a 4-chamber system (each 300 mL volume), custom made to fit the fish (1 kg animal: 10 L water). All chambers were submerged in a larger water bath (139 × 52 × 20 cm), where temperature and oxygen levels were controlled and set to match experimental treatments. A closed recirculation loop pumped low flowing water over the fish in individual chambers. To reduce background respiration, water was pumped through a heater/chiller unit fitted with a UV lamp to sterilize the water. Further, the whole system was rinsed every third day to ensure that background consumption of oxygen remained below 15% of the resting metabolic rate of fish. The remaining chamber was used to record background respiration each day and was randomized each day of recording.

Each chamber was fitted with a fibre optic oxygen probe (FireSting, Pyroscience, OXROB3), which recorded oxygen consumed during each Ṁo_2_ determination (mgO_2_ kg^−1^ h^−1^). A Ṁo_2_ determination period uses the slope of the line of oxygen consumption by fish for each 20 minute determination period before water is replenished to the chamber for 2 minutes (flushing period). Water was circulated intermittently after each Ṁo_2_ determination using a flushing pump connected to all chambers to completely replenish the chamber with oxygenated water from the water bath. During the 20 min determination period, oxygen was not reduced to less than 1 mgO_2_ L^−1^ and was above the background respiration rates. Maximum and standard metabolic rates (MMRs and SMRs) were determined using a modified version of the method described by Roche *et al.* (2013), where fish were chased to exhaustion for 2 min or until fish stopped responding and exposed to the air for 40 sec before being placed inside chambers. For the exhaustive chase, individual fish were placed in a 25 L bucket and encouraged to swim continuously by gently touching the tip of the tail. At the completion of the exhaustive chase, fish were suspended in air in a mesh net for 40 sec and then placed immediately inside a chamber. MMR was measured during the first determination period. Fish were then left in the chamber for ~ 24 hrs to allow them to reach a resting state. The SMR and Ṁo_2_ were calculated for each determination cycle using the equation:

$$\dot{\mathrm{M}}{\mathrm{O}}_2=\Big({\left[{\mathrm{O}}_2\right]}_{t0}-\left[{\mathrm{O}}_2\Big]{}_{t1}\right).\frac{V}{t}.\frac{1}{\mathrm{BW}}$$

where (t0) is the oxygen content (mgO_2_/L) of the water at the conclusion of a flushing cycle and (t1) is the oxygen content measured at the end of a determination period, prior to the next flushing cycle. *V* is the volume of the chamber minus the volume of the experimental animal in *L*, *t* is t0−t1 and BW is the body weight of the experimental animal in kg. The lowest 10% of measurements were averaged to calculate SMR. Background rates were subtracted from Ṁo_2_ values upon calculation. The absolute aerobic scope of fish was calculated by subtracting SMR from MMR (MMR-SMR).

### Determining tolerance to low-oxygen and critical oxygen tension (*P*_crit_)

In order to record tolerance limits of fish amongst the different treatments, we left fish in chambers with the intermittent flushing cycle turned off with only access to the oxygen available from water in the chamber (closed respirometry). Fish were observed constantly during this period. Fish reached a low-oxygen tolerance limit when they lost equilibrium, at which point oxygen level in mgO_2_ L^−1^ was recorded and fish were immediately removed from the chamber. The critical oxygen tension or *P*_crit_ of fish was measured using data from this closed respirometry phase. *P*_crit_ was defined as the point at which Ṁ_O2_ was reduced below SMR and fish shifted to an oxy-conforming state. *P*_crit_ was determined for each fish by fitting a segmented regression using RStudio Version 1.1.419 (segmented package, https://cran.r-project.org/web/packages/segmented/segmented.pdf), a method adapted from Yeager and Ultsch (1989) and Cook *et al.* (2013). The critical tension was recorded as the point of intersection of the two lines as this indicated the breakpoint at which oxy-regulating individuals changed to oxy-conforming individuals. This measure differed from the low-oxygen tolerance point as it occurred prior to fish losing equilibrium.

### Statistical analyses

A linear mixed effects model was fit to the experimental data for MMR, SMR, AAS, *P*_crit_ and loss of equilibrium using the R-package lmerTest (Kuznetsova *et al.* 2017). Factors included temperature and oxygen for Experiment 1 and temperature and acclimation duration for Experiment 2. Prior to model fitting, the distribution of the response variable was inspected using quantile comparison plots. Each of the response variables closely followed a normal distribution, and hence this was considered the most appropriate distribution to model the data and warranted the use of the linear mixed effects model. All treatment levels of temperature, oxygen (normoxic or low-oxygen) and acclimation duration (7, 14 or 30 days) were treated as fixed factors in both experiments. Post-hoc pairwise tests were conducted using the least squares means of the fixed effects where significant effects of the fixed factors were evident in the linear mixed effects model. Tank was treated as a random effect. To assess the variance component of the models, the residuals were plotted against the fitted values. The random scatter observed around zero indicated constant variance across the fitted values for each model. All model analyses were undertaken using R-Studio Version 1.1.419 (R Core Team 2018).

Rearing water temperature, dissolved oxygen (DO) and oxygen saturation in the experimental tanks, as well as Fulton’s K factor, were analysed at all possible treatment levels (temperature, oxygen and acclimation duration) using a 2-factor permutational univariate analysis of variance (ANOVA) with unrestricted permutations using PRIMER 6 & PERMANOVA+ software (www.primer-e.com). All PERMANOVA+ analyses included Monte Carlo permutation tests to derive the probability value and ensure there were sufficient permutations to detect significant differences in all tests. All PERMANOVA+ statistical analyses were initially conducted as 3-factor permutational univariate ANOVAs with tank as the third factor treated as a random factor nested in temperature, oxygen or acclimation duration dependent on the experiment. No effects of tank were detected for rearing water or during any of the experimental responses in either experiment; therefore, data were pooled and 2-factor permutational univariate ANOVAs were conducted removing tank as a factor.

## Results

### Experiment 1: effects of temperature and low-oxygen on fish physiology after 30 days exposure

#### Metabolic scope

**Figure 1:**
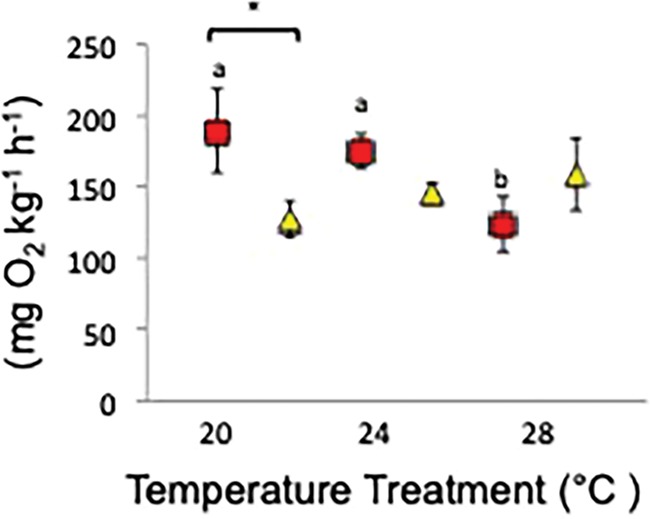
Mean (±SE) SMR for individual Murray cod at all temperatures (20, 24 and 28°C) and treatments (low-oxygen, 3–4 mgO_2_ L^−1^ or 7–9 kPa, and normoxia, 6–8 mgO_2_ L^−1^ or 12–14 kPa), replicate tanks were pooled. Multifactorial pairwise comparisons are indicated by letters and asterisks where significant differences occur (*P* < 0.05). Letters indicate significant differences occurring amongst temperatures for each oxygen level (normoxic or low-oxygen). Brackets and asterisks indicate significant differences occurring bet-ween oxygen levels for each temperature. Red squares represent low-oxygen, 20°C *n* = 8, 24°C *n* = 11 and 28°C *n* = 9, and yellow tri-angles represent normoxia 20°C *n* = 11, 24°C *n* = 11 and 28°C *n* = 10.

There was an interactive effect between temperature and oxygen treatments on SMR, with fish having higher SMR within the low-oxygen treatments except at the highest temperature (28°C, *P* = < 0.050 Table S3, Fig. 1). SMR was higher in low-oxygen treatments than in normoxic treatments at 20°C (*P* = 0.020, Fig. 1, Table S3). MMR and AAS were not affected by low-oxygen exposure or temperature treatments (Table S3).

#### Low oxygen tolerance limits & *P*_crit_

**Figure 2:**
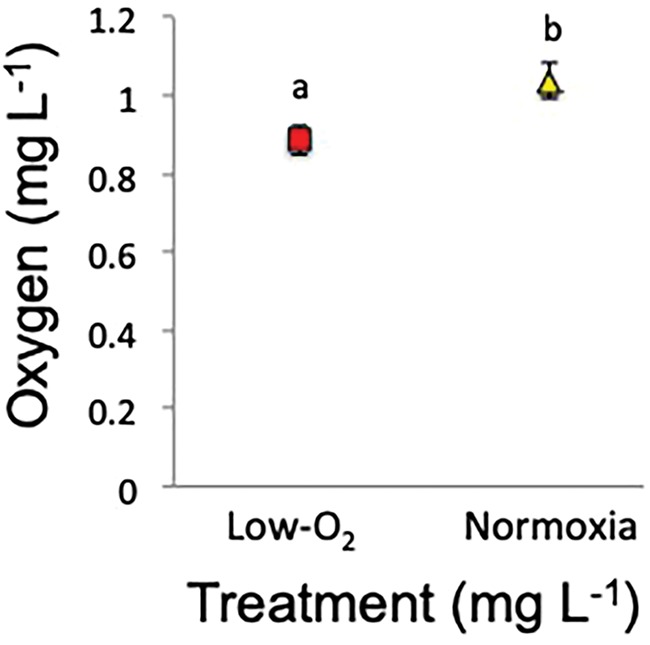
Mean (±SE) low-oxygen tolerance at loss of equilibrium from exposure to low-oxygen or normoxia for individual Murray cod (low-oxygen 3–4 mgO_2_ L^−1^ or 7–9 kPa, *n* = 28 and normoxia 6–8 mgO_2_ L^−1^ or 12–14 kPa, *n* = 34). Multifactorial pairwise comparisons are indicated by letters where significant differences occur (*P* < 0.05). Red squares represent low-oxygen and yellow triangles represent normoxia.

**Figure 3:**
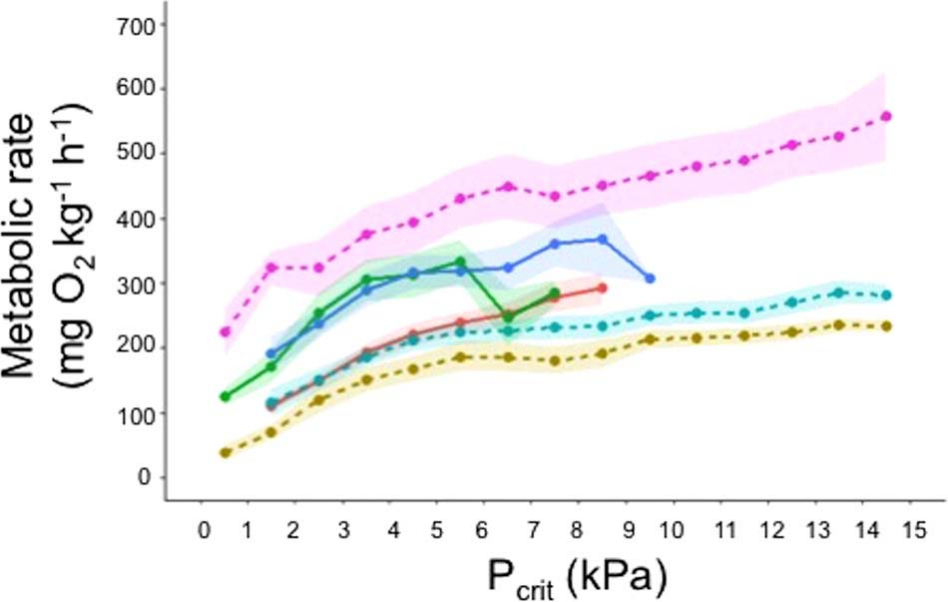
*P*_crit_ and average metabolic rate of Murray cod at different kPas, temperatures (20, 24 and 28°C) and oxygen exposures (low-oxygen, 3–4 mgO_2_ L^−1^ or 7–9 kPa, solid lines or normoxia, 6–8 mgO_2_ L^−1^ or 12–14 kPa, dashed lines). Treatments are distinguished by colour with yellow representing 20°C under normoxia (*n* = 10), red representing 20°C under low-oxygen (*n* = 8), teal representing 24°C under normoxia (*n* = 11), green representing 24°C under low-oxygen (*n* = 11), pink representing 28°C under normoxia (*n* = 9) and blue representing 28°C under low-oxygen (*n* = 9). Shading around the lines indicates standard error.

Fish exposed to low-oxygen maintained equilibrium for longer than those exposed to normoxic conditions (*P* = 0.022, Fig. 2, Table S4). The *P*_crit_ of fish was unaffected by hypoxia or temperature (hypoxia *P* = 0.121 and temperature *P* = 0.332, Table S6, Fig. 3).

### Experiment 2: acclimation of fish at two temperatures under low-oxygen conditions

#### Metabolic scope

**Figure 4:**
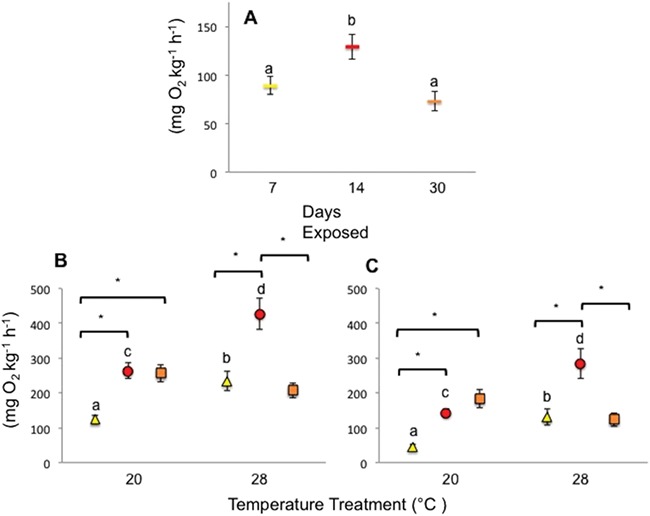
Mean (±SE) (A) AAS for Murray cod exposed to low-oxygen for different acclimation times (7, *n* = 20, 14, *n* = 16 and 30 days, *n* = 17); temperature treatments and replicate tanks have been pooled as no significant effects were detected. (B) MMR and (C) SMR for Murray cod exposed to low-oxygen at 20 and 28°C, for three different acclimation times (7, 14 and 30 days). Shapes and colours have been used to differentiate acclimation times (yellow triangle = 7 days, red circle = 14 days and orange square = 30 days), except in (A) where rectangles were used as temperatures were pooled. Multifactorial pairwise comparisons are indicated by letters and asterisks where significant differences occur (*P* < 0.05). Letters indicate significant differences occurring between temperatures for each acclimation time (7, 14 or 30 days). Brackets and asterisks indicate significant differences occurring amongst acclimation times for each temperature. For both (B) and (C), yellow triangles represent 7 days exposure at 20°C *n* = 11 and 28°C *n* = 9, red circles represent 14 days exposure at 20°C *n* = 10 and 28°C *n* = 6 and orange squares represent 30 days exposure at 20°C *n* = 9 and 28°C *n* = 9.

Murray cod had the highest aerobic capacity (AAS) after 14 days exposure to low-oxygen and the lowest after 30 days exposure (*P* = < 0.001, Fig. 4A, Table S5). No effect of temperature was detected for AAS (*P* = 0.06, Table S5). There was an interaction between temperature and exposure time for both measures of metabolic rate (SMR and MMR) with fish held at 28°C and 14 days exposure to low-oxygen having the highest metabolic rates (*P* = < 0.001, Fig. 4B and C, Table S5). Fish held at 20°C and 7 days exposure had the lowest metabolic rates (*P* = < 0.001, Fig. 4B and C, Table S5). Additionally, fish had higher metabolic rates after 7 and 14 days exposure at 28°C compared to 20°C in both SMR and MMR (*P* = < 0.001, Fig. 4B and C, Table S5).

#### Loss of equilibrium & *P*_crit_

**Figure 5:**
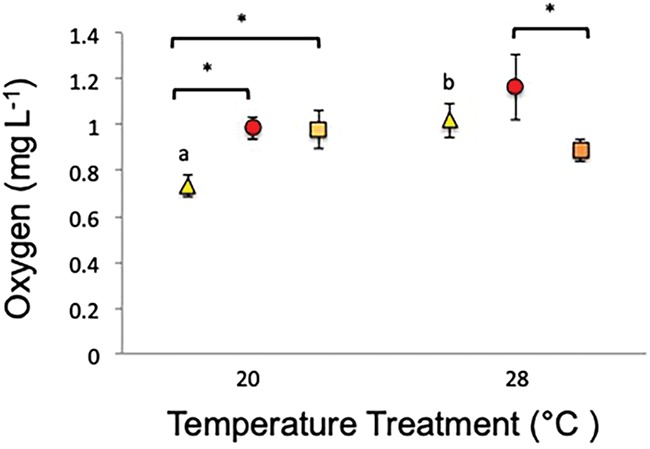
Mean (±SE) low-oxygen tolerance at loss of equilibrium for individual Murray cod exposed to low-oxygen at 20 and 28°C, for three different acclimation times (7, 14 and 30 days). Multifactorial pairwise comparisons are indicated by letters and asterisks where significant differences occur (*P* < 0.05). Letters indicate significant differences between temperatures for each acclimation time (7, 14 or 30 days). Brackets and asterisks indicate significant differences occurring amongst the three acclimation times for each temperature. Yellow triangles represent 7 days exposure at 20°C *n* = 11 and 28°C *n* = 9, red circles represent 14 days exposure at 20°C n = 13 and 28°C *n* = 6 and orange squares represent 30 days exposure at 20°C *n* = 9 and 28°C *n* = 9

Fish exhibited the greatest tolerance to low-oxygen at 20°C after only 7 days exposure; however, fish held at 28°C had the greatest tolerances after 30 days exposure to low-oxygen (post-hoc; between 20 & 28°C after 7 days, *P* = 0.005, and between different acclimation times at 20°C, 7 & 14 days, *P* = 0.006 and 7 & 30 days, *P* = 0.013 and at 28°C between 14 & 30 days, *P* = 0.019; Fig. 5, Table S6). The *P*_crit_ of fish was unaffected by acclimation time or temperature (acclimation time *P* = 0.160 and temperature *P* = 0.376, Table S6).

#### Fish condition

Fish condition (Fulton’s *K*) was lowest at 28°C after exposure to low-oxygen for one month (post-hoc; after exposure to low-oxygen between 20 & 28°C *P* = 0.001 and 24 & 28°C *P* = 0.001; Table S7, Fig. S1A). During the acclimation trials, fish exposed to low-oxygen for 7 days were healthier (had a higher Fulton’s K) than fish exposed for longer (Post-hoc; between 7 & 14 days *P* = 0.001 and 7 & 30 days *P* = 0.001), and fish reared at 20°C fared much better during all acclimation periods than those reared at 28°C (*P* = 0.001; Table S7, Fig. S1B).

## Discussion

Our results show that prior exposure to low-oxygen could improve the tolerance of fish to hypoxic conditions. Additionally, we showed that time spent under low-oxygen conditions impacted physiological performance. However, counter to expectations, physiological performance (aerobic ability) did not improve with exposure to low-oxygen compared with fish exposed to normoxia.

Prolonged exposure to low-oxygen improved the tolerance of fish relative to those exposed to normoxia, allowing them to tolerate lower levels of dissolved oxygen before losing equilibrium. Additionally, acclimation time influenced low-oxygen tolerance such that fish exposed for greater periods of time had poorer tolerance, though critical oxygen tension (*P*_crit_) remained unaffected. However, the difference between average loss of equilibrium after low-oxygen exposure in our treatments was small compared to normoxic treatments (<1 mg L^−1^), suggesting that it may not equate to a significant response in the wild. Acclimation of fish to hypoxia may be a naturally selected trait for species living in areas prone to hypoxia. For example, acclimation to seasonal hypoxia and diel hypoxia exposure has improved the tolerance of a number of fish species (Collins *et al.* 2016; McBryan *et al.* 2016; Rogers *et al.* 2016). However, chronic and daily exposure over long time periods does not always result in improved tolerance particularly in sensitive fish species (Cook *et al.* 2013; Remen *et al.* 2013). Our results suggest that prior exposure to low-oxygen conditions may improve tolerance of fish to hypoxia but that prolonged acclimation time to those conditions may significantly reduce survival. Multiple studies have found unique adaptations to hypoxia based on life history and habitat use (McBryan *et al.* 2016). Species which experience a greater fluctuation of environmental conditions such as those inhabiting temperate, estuarine and freshwater systems are likely to have a higher level of plasticity than species which remain in stable/slow-changing environments. Freshwater species may well have improved hypoxia tolerance due to more frequent exposure than saltwater species; this has been supported by differences in *P*_crit_ between the two groups (Rogers *et al.* 2016). Our study species, Murray cod, has a broad geographical distribution and likely adapted to a wide range of thermal and hypoxic conditions, and this study provides new hypoxia data for a species of conservation priority (for further species information, see SI; Koehn and Nicol 2016).

As ectotherms, stress created through temperature changes directly affects growth and metabolic rate (Neuheimer *et al.* 2011). Combined low oxygen and high temperatures can be physiologically challenging, making mild hypoxic conditions potentially lethal at higher temperatures (McBryan *et al.* 2016; Sinclair *et al.* 2016). Prolonged low-oxygen exposure improved resting oxygen requirements (SMR) at 28°C compared to the normoxic control in our study, suggesting an acclimation response however; other metabolic rate measures at the same temperature were unaffected. Fish responses to changes in temperature and hypoxia vary significantly amongst and within species (Pörtner and Farrell 2008; Healy and Schulte 2012; Sandblom *et al.* 2014; Rogers *et al.* 2016). Gill remodelling to increase gill surface area has improved oxygen uptake in response to temperature and hypoxia for some species (Sollid *et al.* 2005; McBryan *et al.* 2016). Other influences which may impact hypoxia tolerance include: variation in oxygen consumption influenced by ATP production, oxygen-carrying capacity of blood and environmental influences such as changes to food intake, diet composition and ambient conditions (Salin *et al.* 2015; Collins *et al.* 2016; Rogers *et al.* 2016). Prolonged exposure to low-oxygen at the lower end of the species thermal range reduced aerobic capacity. Additionally, varied effects of temperature were observed after exposure to hypoxia for different durations under measures of metabolic capacity of Murray cod. In contrast, a study that investigated thermal acclimation in Murray cod found that they were temperature-independent, such that they had a greater capacity to transport oxygen to tissues regardless of higher temperatures (Clark *et al.* 2005). Aerobic scope values in our study appeared to be similar to aerobic scope values observed by Clark *et al.* (2005), however, our results did not indicate that Murray cod was temperature-independent. The difference in our findings regarding temperature may be explained by a difference in method as Clark *et al.* (2005) investigated swimming respirometry rates under a higher flow, while we investigated resting rates of this species, as well as exposing fish for a prolonged period to low-oxygen conditions. Declines in hypoxia tolerance have been attributed to elevated temperatures raising metabolic demands in some species (McBryan *et al.* 2016), however; multiple species have displayed improved tolerance to hypoxia following temperature stress (Todgham *et al.* 2005; Burleson and Silva 2011; McBryan *et al.* 2013; Fu *et al.* 2014). Fitness of other species has also been shown to decline due to repeated exposure to high temperatures; this is particularly prominent in lizards and insects (Bickler and Buck 2007).

Activity profiles of organisms (i.e. active versus sedentary lifestyles) are associated with contrasting levels of aerobic capacity, such that there is a trade-off for locomotive performance and tolerance to low resource availability, in particular oxygen (Killen *et al.* 2016). Species better adapted to low levels of oxygen have lower aerobic capacity and are able to initiate changes to increase oxygen extraction and transport by adjusting gill surface area, oxygen affinity of haemoglobin and muscle mitochondrial density (Nilsson and Ostlund-Nilsson 2008; Killen *et al.* 2016). Prolonged exposure to low-oxygen improved the tolerance of Murray cod, although in nature this may have had minimal impact on hypoxic tolerance and was not reflected in our metabolic tests. Aerobic ability of fish was linked to long-term low-oxygen exposure, such that fish exposed for a longer period did not show any marked improvement in low-oxygen tolerance and only showed improvement in aerobic ability after 14 days of acclimation. Therefore, resistance to hypoxia may be likely to decrease as a function of exposure time. Furthermore, our results suggest that the duration of low-oxygen exposure may play an important role in hypoxia tolerance and post-hypoxic exposure metabolism. The lack of distinct effects of temperature and low-oxygen on the metabolic rate and aerobic scope could still indicate acclimation ability. Another study showed that temperature dramatically reduced tolerance to hypoxia, even when aerobic scope was minimally affected (McBryan *et al.* 2013). Acclimation responses of fish remain largely unknown. Fish may move and encounter areas with low oxygen, but can actively avoid them (except during widespread events), thereby not acclimating to those conditions, leaving them less likely to survive future hypoxic events. Transgenerational transfers of hypoxia tolerance amongst individuals are also evident in some species (Rogers *et al.* 2016). Acclimation to hypoxic conditions may not be viable for all species as some may be unable to make selective trade-offs to cope with a changing climate. Understanding physiological responses of fish to environmental stressors is crucial for predicting future ecological impacts between environmental change and population level effects which will aid in setting conservation targets (Rogers *et al.* 2016).

Acclimation to hypoxia is likely to be accompanied by changes to the oxygen transport capacity of blood (Collins *et al.* 2016; Rogers *et al.* 2016). Some species exhibit increased haemoglobin and haematocrit after chronic hypoxia exposure resulting in improved tolerance to hypoxia (Collins *et al.* 2016; Rogers *et al.* 2016); however, tolerance to hypoxia does not always change (Collins *et al.* 2016; Rogers *et al.* 2016). In our study, acclimation of fish at higher temperatures could have been improved by increases in haemoglobin leading to increased oxygen-carrying capacity of the blood improving oxygen uptake during chronic exposure. Increases in temperature reduce haemoglobin oxygen-binding affinity, as the haemoglobin molecule is thermally sensitive; therefore, acclimation of fish to higher temperatures could counteract reduced transport efficiency by increasing the amount of oxygen picked up at the gills (McBryan *et al.* 2016). Increased transport of haemoglobin may explain the possible acclimation of our species at the highest temperature after the longest acclimation time. Manipulation of the oxygen-carrying capacity of blood in relation to hypoxia and temperature is likely to be species-specific and may be affected by life-history traits and the nature of the hypoxic event (Collins *et al.* 2016). Therefore, future research would benefit from testing the oxygen-carrying capacity of haemoglobin when investigating hypoxia tolerance.

Progressive warming and an increased propensity for hypoxic events in aquatic environments are of critical conservation concern for fish as these stressors are associated with shifts in phenology, distribution, abundance and reproduction, as well as large-scale mortalities (Breitburg *et al.* 2009; Norin *et al.* 2014; Rogers *et al.* 2016). Riverine ecosystems have been largely degraded throughout the world, due to flow mismanagement and the construction of barriers that limit fish movements (Dwyer *et al.* 2014; Small *et al.* 2014; Koehn and Nicol 2016). For example, the Murray–Darling basin is home to 46 native species, including Murray cod, but only represents 10% total abundance of their pre-European settlement populations (Koehn and Nicol 2016). To manage the effects of combined temperature and hypoxia, we need to understand the relationship between instantaneous physiological performance (the focus of most physiologically targeted studies) and long-term fitness as well as post-hypoxic exposure responses (Sinclair *et al.* 2016). Physiological information on species hypoxia tolerance and overall aerobic capacity can be incorporated into models to predict the long-term outcomes of deliberate water releases and natural flooding events on aquatic life. Models have successfully predicted changes in populations due to different stressors, and there is a growing trend to incorporate multi-species data into models to maximize the benefits of future conservation management plans (Sherman *et al.* 2007; Koehn and Nicol 2014). Data, which could be incorporated to aid model efficiency, include longitudinal studies in nature or molecular and physiological markers of performance (Sinclair *et al.* 2016). Presently, a number of management actions exist for the recovery of Murray cod such as stock enhancements, translocation efforts, habitat rehabilitation, legislative protection, remediation of barriers to fish passage, improved water quality and flow management and control of alien species (Lintermans 2013). However, if hypoxic events cannot be controlled or managed, these efforts will provide little relief for this iconic species. Our study showed that Murray cod could persist in low-oxygen conditions, particularly after prior exposure, with temperature having minimal effect on physiological response. However, prolonged exposure to low-oxygen conditions may reduce long-term survival after greater duration periods. By informing water managers, we can aid in meeting conservation conditions for species like Murray cod. For example, environmental water flows could be controlled for release to alleviate low-oxygen conditions that persist for longer than 14 days, which may impact Murray cod.

Aerobic scope measures alone were not sufficient in explaining hypoxia acclimation in our study. Conclusions based on oxygen consumption rates alone (metabolic scope) could thus lead to erroneous interpretations about the acclimation abilities of fish when faced with environmental stressors such as hypoxia and elevated temperatures. Behavioural tests on the tolerance of fish to low-oxygen illustrated the possible acclimation ability of Murray cod, in particular how prolonged exposure to low-oxygen conditions may physiologically reduce tolerance long-term. Other species may be similarly affected by prolonged periods of low-oxygen conditions, and our results provide much needed hypoxia data on a species of conservation concern. Future research should target numerous species and their ability to acclimate to hypoxia in combination with other stressors. In particular, species recovery from hypoxia has been largely overlooked and will aid in understanding the capabilities of fish to not only withstand but also endure hypoxic events. Furthermore, development of a universal method to measure acclimation response to hypoxia exposure would allow direct comparisons amongst different species as research in this field continues.

## Supplementary Material

SuppInfoEdited_coz058
