# Supplementary material for: Prolonged exposure to low oxygen improves hypoxia tolerance in a freshwater fish
Source: Conserv Physiol. 2019 Nov 28;7(1):coz058. doi: 10.1093/conphys/coz058 (PMC6882409; doi:10.1093/conphys/coz058)
Supplement: SuppInfoEdited_coz058 [file suppinfoedited_coz058.docx]

SUPPLEMENARY DATA:

**Supplementary Information 1:**

**Study Species**

Percichthyidae: *Maccullochella peelii* (Murray cod)

Murray cod is Australia’s largest solely freshwater fish reaching sizes of 1800mm in length with weights recorded up to 113.6kg. It reaches maturity at around 4-5 years for both sexes (Couch *et al.* 2016). This species occurs throughout the Murray-Darling system, a system that encompasses 5 separate states and is the largest catchment in Australia covering approx. 1 million km^2^ (Koehn and Nicol 2016). At present there are active national recovery programs in place for the conservation of this species which is listed as vulnerable under the *Environmental Protection and Biodiversity Conservation* Act 1999 (EPBC) and as critically endangered on the IUCN Red List (www.iucnredlist.org, (Couch *et al.* 2016). This species has a predominantly sedentary lifestyle, and commonly uses a sit-and-wait predation technique, but is also well known for making large migrations (>1km (Clark *et al.* 2005). Murray cod inhabit a wide variety of habitats including clear, rocky streams to slow flowing, turbid rivers and billabongs, with a strong preference for structural woody habitats (Koehn and Nicol 2016). Their abundance has been drastically reduced across their natural range due to both natural (e.g. increased temperature, hypoxia) and anthropogenic factors (e.g. extensive barriers in place which limit fish passage and environmental watering (Koehn and Nicol 2016).

**SUPP Table 1.** Summary of the rearing conditions and length and weight data of the fish within treatment tanks for physiological experiments. Data are displayed as means ± standard error (SE) with *n* representing the sample size of fish. For temperature, dissolved oxygen and saturation, *n* denotes the number of recordings taken throughout the experimental period; this differed with exposure length.

| **Desired Treatments** | Oxygen, mg L^-1^ | Tank | Temperature, °C | Dissolved Oxygen,  mg L^-1^ | Saturation, % | Fish Length, cm | Fish Weight, g | *n* |
| --- | --- | --- | --- | --- | --- | --- | --- | --- |
| **7 Days Exposure** |  |  | (*n*=5) | (*n*=5) | (*n*=5) |  |  |  |
|  |  |  |  |  |  |  |  |  |
| 20 | 4 | 1 | 20.0±0.09 | 3.92±0.02 | 56.4±0.26 | 5.7±0.19 | 2.3±0.18 | 6 |
|  |  | 2 | 20.2±0.07 | 3.96±0.01 | 57.2±0.09 | 5.9±0.08 | 2.4±0.23 | 5 |
| 28 | 4 | 1 | 28.0±0.14 | 3.96±0.02 | 53.6±0.15 | 6.3±0.23 | 2.2±0.23 | 6 |
|  |  | 2 | 28.3±0.05 | 3.88±0.01 | 55.4±0.13 | 5.8±0.23 | 2.2±0.18 | 3 |
|  |  |  |  |  |  |  |  |  |
| **14 Days Exposure** |  |  | (*n*=9) | (*n*=9) | (*n*=9) |  |  |  |
|  |  |  |  |  |  |  |  |  |
| 20 | 4 | 1 | 19.9±0.10 | 3.80±0.01 | 51.0±0.31 | 5.8±0.11 | 2.0±0.16 | 7 |
|  |  | 2 | 19.8±0.03 | 3.87±0.01 | 53.5±0.12 | 5.7±0.15 | 1.8±0.19 | 6 |
| 28 | 4 | 1 | 28.2±0.04 | 3.94±0.01 | 54.5±0.11 | 5.1±0.01 | 1.4±0.01 | 2 |
|  |  | 2 | 27.8±0.04 | 3.90±0.01 | 56.4±0.12 | 5.6±0.16 | 1.1±0.21 | 5 |
|  |  |  |  |  |  |  |  |  |
| **30 Days Exposure** |  |  | (*n*=17) | (*n*=17) | (*n*=17) |  |  |  |
|  |  |  |  |  |  |  |  |  |
| 20 | 4 | 1 | 20.4±0.08 | 4.07±0.01 | 50.0±0.15 | 6.1±0.02 | 2.2±0.16 | 5 |
|  |  | 2 | 20.1±0.04 | 3.95±0.01 | 52.5±0.09 | 5.8±0.06 | 1.9±0.16 | 3 |
|  | 8 | 1 | 20.2±0.06 | 6.81±0.01 | 90.6±0.08 | 6.0±0.10 | 1.6±0.06 | 6 |
|  |  | 2 | 20.9±0.04 | 6.75±0.01 | 91.3±0.07 | 5.8±0.18 | 1.9±0.17 | 5 |
| 24 | 4 | 1 | 24.3±0.04 | 3.79±0.01 | 53.6±0.07 | 5.6±0.05 | 1.6±0.10 | 5 |
|  |  | 2 | 24.4±0.03 | 3.94±0.01 | 56.8±0.06 | 6.1±0.16 | 2.4±0.18 | 6 |
|  | 8 | 1 | 24.2±0.04 | 6.78±0.01 | 89.4±0.06 | 6.2±0.24 | 2.1±0.18 | 5 |
|  |  | 2 | 24.1±0.03 | 6.85±0.01 | 92.1±0.04 | 5.9±0.12 | 1.8±0.18 | 6 |
| 28 | 4 | 1 | 28.0±0.03 | 3.89±0.01 | 57.4±0.06 | 6.7±0.17 | 2.3±0.18 | 5 |
|  |  | 2 | 28.2±0.04 | 3.78±0.01 | 55.5±0.06 | 6.6±0.19 | 2.1±0.18 | 4 |
|  | 8 | 1 | 28.3±0.03 | 6.90±0.01 | 92.2±0.06 | 5.5±0.06 | 1.3±0.05 | 4 |
|  |  | 2 | 27.9±0.03 | 6.58±0.01 | 91.5±0.05 | 5.5±0.13 | 1.4±0.13 | 8 |

**SUPP Table 2.** Analysis of variance examining the effects of measured temperature (temp), dissolved oxygen (DO), and oxygen saturation (SAT) on temperature and low oxygen treatments in the rearing water for the month long exposure and acclimation to low oxygen. * refers to P values <0.05

| **Water** | Source of Variation | df | MS | F | P |
| --- | --- | --- | --- | --- | --- |
| **Month long exposure** |  |  |  |  |  |
| **Temp** | Temp | 2 | 1013.3 | 15427 | ≤0.001* |
|  | Oxygen | 1 | 0.14 | 2.18 | >0.050 |
|  | Temp X Oxygen | 2 | 1.29 | 19.69 | ≤0.001* |
|  | Residuals | 198 | 6.5x10^2^ |  |  |
| **DO** | Temp | 2 | 0.21 | 26.08 | ≤0.001* |
|  | Oxygen | 1 | 420.8 | 53053 | ≤0.001* |
|  | Temp X Oxygen | 2 | 0.15 | 18.35 | ≤0.001* |
|  | Residuals | 198 | 7.9x10^3^ |  |  |
| **SAT** | Temp | 2 | 158.9 | 120.49 | ≤0.001* |
|  | Oxygen | 1 | 69446 | 52656 | ≤0.001* |
|  | Temp X Oxygen | 2 | 97.34 | 73.81 | ≤0.001* |
|  | Residuals | 198 | 1.32 |  |  |
| **Acclimation to low oxygen** |  |  |  |  |  |
| **Temp** | Temp | 1 | 1560.4 | 28298 | ≤0.001* |
|  | Days Exposed | 2 | 0.55 | 9.91 | ≤0.001* |
|  | Temp X Days Exposed | 2 | 0.13 | 2.35 | >0.050 |
|  | Residuals | 118 | 5.5x10^2^ |  |  |
| **DO** | Temp | 1 | 4.1x10^2^ | 11.99 | ≤0.001* |
|  | Days Exposed | 2 | 2.6x10^2^ | 7.69 | ≤0.001* |
|  | Temp X Days Exposed | 2 | 0.20 | 58.58 | ≤0.001* |
|  | Residuals | 118 | 3.5x10^3^ |  |  |
| **SAT** | Temp | 1 | 99.59 | 72.76 | ≤0.001* |
|  | Days Exposed | 2 | 26.33 | 19.23 | ≤0.001* |
|  | Temp X Days Exposed | 2 | 106.86 | 78.07 | ≤0.001* |
|  | Residuals | 118 | 1.37 |  |  |

**SUPP Table 3.** Linear mixed effects model examining the effects of temperature and oxygen on the metabolic rates of Murray cod. All possible temperatures (20, 24 and 28°C) and oxygen treatments (normoxic 6-8 and low oxygen 3-4mg L^-1^) are considered for fish exposed to treatments for 30 days.

| **Metabolic Rate** | Sources of Variation | df | MS | F | P |
| --- | --- | --- | --- | --- | --- |
| **MMR** | Temp | 2 | 3451.9 | 0.71 | >0.050 |
|  | Oxygen | 1 | 1996.1 | 0.41 | >0.050 |
|  | Temp X Oxygen | 2 | 4580.4 | 0.95 | >0.050 |
|  |  |  |  |  |  |
| **SMR** | Temp | 2 | 2168.5 | 0.70 | >0.050 |
|  | Oxygen | 1 | 5039.2 | 1.63 | >0.050 |
|  | Temp X Oxygen | 2 | 11468 | 3.71 | <0.050* |
|  |  |  |  |  |  |
| **AAS** | Temp | 2 | 159.1 | 0.08 | >0.050 |
|  | Oxygen | 1 | 5107.0 | 2.61 | >0.050 |
|  | Temp X Oxygen | 2 | 3017.8 | 1.54 | >0.050 |

** Denotes significant difference.*

**SUPP Table 4.** Linear mixed effects model examining the effects of temperature and oxygen on the ability of Murray cod to tolerate low-oxygen conditions. All possible temperatures (20, 24 and 28°C) and oxygen treatments (normoxic 6-8 and low oxygen 3-4, mg L^-1^) are considered.

| **Low-oxygen Tolerance** | Sources of Variation | df | MS | F | P |
| --- | --- | --- | --- | --- | --- |
| **Pcrit** | Temp | 2 | 2.57 | 1.39 | >0.050 |
|  | Oxygen | 1 | 6.44 | 3.49 | >0.050 |
|  | Temp X Oxygen | 2 | 5.24 | 2.84 | >0.050 |
|  |  |  |  |  |  |
| **Loss of equilibrium** | Temp | 2 | 0.01 | 0.27 | >0.050 |
|  | Oxygen | 1 | 0.33 | 5.57 | <0.050* |
|  | Temp X Oxygen | 2 | 0.05 | 0.88 | >0.050 |

** Denotes statistical significance*

**SUPP Table 5.** Linear mixed effects model for the effects of temperature and length of exposure (7,14 or 30 days) to low oxygen on the metabolic rates of Murray cod. All possible temperatures (20 and 28°C) are considered.

| **Metabolic Rate** | Sources of Variation | df | MS | F | P |
| --- | --- | --- | --- | --- | --- |
| **MMR** | Temp | 1 | 73412 | 13.94 | ≤0.001* |
|  | Days Exposed | 2 | 120506 | 22.88 | ≤0.001* |
|  | Temp X Days | 2 | 52306 | 9.93 | ≤0.001* |
|  |  |  |  |  |  |
| **SMR** | Temp | 1 | 40165 | 10.19 | ≤0.001* |
|  | Days Exposed | 2 | 67884 | 17.22 | ≤0.001* |
|  | Temp X Days | 2 | 46046 | 11.68 | ≤0.001* |
|  |  |  |  |  |  |
| **AAS** | Temp | 1 | 7157.3 | 3.73 | >0.050 |
|  | Days Exposed | 2 | 14621.8 | 7.62 | ≤0.001* |
|  | Temp X Days | 2 | 10.6 | 0.005 | >0.050 |

** Denotes significant difference.*

**SUPP Table 6.** Linear mixed effects model for the effects of temperature and days exposure (7, 14 or 30 days) on the ability of Murray cod to tolerate low-oxygen conditions. All possible temperatures (20, 24 and 28°C) are considered.

| **Low-oxygen Tolerance** | Sources of Variation | df | MS | F | P |
| --- | --- | --- | --- | --- | --- |
| **Pcrit** | Temp | 1 | 1.49 | 0.62 | >0.050 |
|  | Days Exposed | 2 | 1.75 | 0.73 | >0.050 |
|  | Temp X Days | 2 | 0.65 | 0.27 | >0.050 |
|  |  |  |  |  |  |
| **Loss of equilibirum** | Temp | 1 | 0.21 | 4.5 | <0.050* |
|  | Days Exposed | 2 | 0.18 | 3.95 | <0.050* |
|  | Temp X Days | 2 | 0.17 | 3.78 | <0.050* |

** Denotes statistical significance*

**SUPP Table 7.** Analysis of variance for the effects of oxygen exposure over a month and acclimation to low oxygen on Fulton’s K condition factor. Month long exposure (Experiment 1) concerns fish exposed to either low oxygen or normoxia for 30 days under three different temperatures (20, 24 and 28°C). Acclimation to low oxygen (Experiment 2) concerns fish exposed only to low oxygen conditions for differing numbers of days (7, 14 and 30 days) and under two different temperatures (20 and 28°C).

| **Fulton’s K Condition Index** | Sources of Variation | df | MS | F | P |
| --- | --- | --- | --- | --- | --- |
| **Exp 1. Temperature** | Temp | 2 | 0.26 | 9.16 | ≤0.001* |
|  | Oxygen | 1 | 3.26x10^2^ | 2.32 | >0.050 |
|  | Temp X Oxygen | 2 | 6.88x10^2^ | 4.89 | ≤0.001* |
|  | Residuals | 56 | 1.41x10^2^ |  |  |
|  |  |  |  |  |  |
| **Exp 2. Acclimation** | Temp | 1 | 0.97 | 37.57 | ≤0.001* |
|  | Days Exposed | 2 | 0.32 | 12.33 | ≤0.001* |
|  | Temp X Days Exposed | 2 | 7.62x10^3^ | 0.29 | >0.050 |
|  | Residuals | 50 | 2.59x10^2^ |  |  |


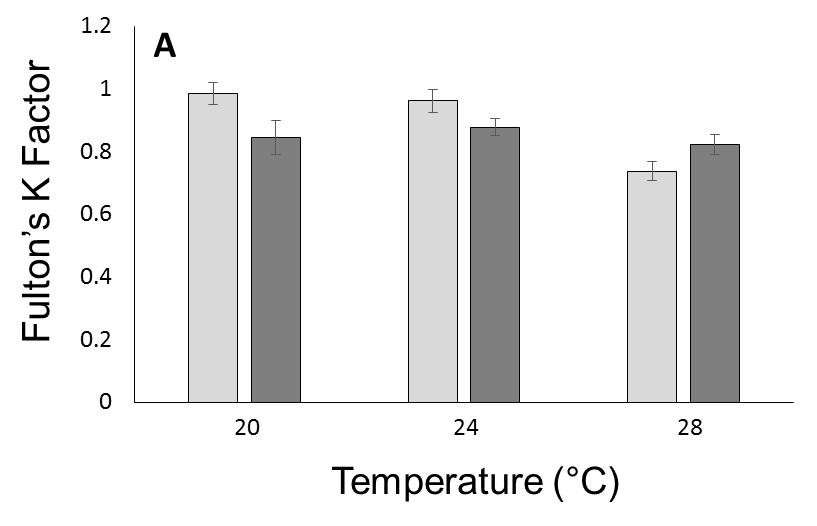

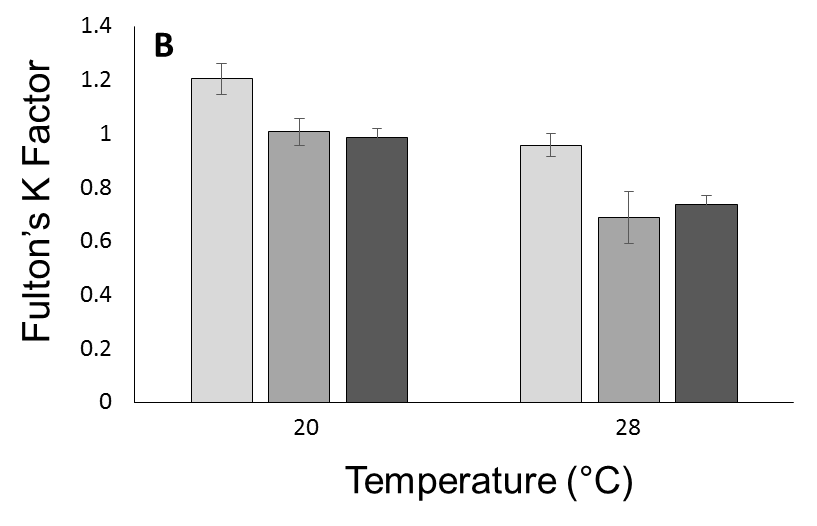


**Figure SUPP 1.** Mean (±SE) Fulton’s K factor A) temperature experiment with exposure to low oxygen (light grey) or normoxia (grey) separated by temperature treatments, and B) after acclimation to low oxygen at two temperatures after 7 (light grey), 14 (grey) or 30 days (dark grey) (n=54).

REFERENCES

Clark, T., D, T. Ryan, B. Ingram, A, A. Woakes, J, P. Butler, J, P. Frappell and B (2005). Factorial aerobic scope is independent of temperature and primarily modulated by heart rate in exercising Murray Cod (*Maccullochella peelii peelii*). Physiological and Biochemical Zoology: Ecological and Evolutionary Approaches **78**(3): 347-355.

Couch, A. J., P. J. Unmack, F. J. Dyer and M. Lintermans (2016). Who’s your mama? Riverine hybridisation of threatened freshwater Trout Cod and Murray Cod. PeerJ **4**: e2593.

Koehn, J. D. and S. J. Nicol (2016). Comparative movements of four large fish species in a lowland river. Journal of Fish Biology **88**(4): 1350-1368.
